# New Vehicle for Holding Camphor in Solution

**Published:** 1848-09

**Authors:** 


*New Vehicle for holding Camphor in Solution.*—Sir James Murray proposes a new vehicle for holding Camphor in solution, which may be exhibited in doses considerably greater, and with less irritation, than it has hitherto been given. It was known that camphor was insoluble in water, and that when given in almond emulsion it very readily separates on the addition of water, and that the same separation takes place on adding water to a solution of camphor in spirits of wine. The opinions respecting the effect of camphor are various. Some describe it as a stimulant, and some as a sedative; but this difference of effect depends mainly on the quantity given. Now, Sir James Murray has found that the fluid magnesia was capable of dissolving camphor to the extent of three grains to the ounce of the solution, and that adding water to the mixture did not cause any cloudiness or separation of the camphor. An ounce of this solution contains three grains of camphor, which appears perfectly clear, like water, and if anything is added to the solution capable of withdrawing a portion of the water, such as dry common salt, a rough estimate may be formed of the quantity of camphor which it contains. To employ camphor as a sedative, it must be given in large doses; but it is also necessary to have it perfectly dissolved, for when free it acts as a powerful stimulant. It is obvious, then, that given for this purpose it would not do to employ the camphorated spirit, nor will the solution in emulsion be any better, as it readily separates from it in the stomach. We have, therefore, he obesrves, a menstruum in the fluid magnesia, which answers better than any method hitherto known.—*Dublin Medical Press.*

